# Identification of a new plant extract for androgenic alopecia treatment using a non-radioactive human hair dermal papilla cell-based assay

**DOI:** 10.1186/s12906-016-1004-5

**Published:** 2016-01-21

**Authors:** Ruchy Jain, Orawan Monthakantirat, Parkpoom Tengamnuay, Wanchai De-Eknamkul

**Affiliations:** 1 Department of Pharmaceutical Technology, Faculty of Pharmaceutical Sciences, Chulalongkorn University, Bangkok, Thailand; 2 Research Unit of Natural Product Biotechnology, Faculty of Pharmaceutical Sciences, Chulalongkorn University, Bangkok, Thailand; 3 Division of Pharmaceutical Chemistry, Faculty of Pharmaceutical Sciences, Khon Kaen University, Khon Kaen, Thailand; 4 Department of Pharmacognosy and Pharmaceutical Botany, Faculty of Pharmaceutical Sciences, Chulalongkorn University, Bangkok, Thailand

**Keywords:** Androgenic alopecia, 5*α*-reductase inhibitory activity, Human hair dermal papilla cell-based assay, Anti-androgenic activity, *Avicennia marina*

## Abstract

**Background:**

Androgenic alopecia (AGA) is a major type of human scalp hair loss, which is caused by two androgens: testosterone (T) and 5*α*-dihydrotestosterone (5*α*-DHT). Both androgens bind to the androgen receptor (AR) and induce androgen-sensitive genes within the human hair dermal papilla cells (HHDPCs), but 5*α*-DHT exhibits much higher binding affinity and potency than T does in inducing the involved androgen-sensitive genes. Changes in the induction of androgen-sensitive genes during AGA are caused by the over-production of 5*α*-DHT by the 5*α*-reductase (5*α*-R) enzyme; therefore, one possible method to treat AGA is to inhibit this enzymatic reaction.

**Methods:**

RT-PCR was used to identify the presence of the 5*α*-R and AR within HHDPCs. A newly developed AGA-relevant HHDPC-based assay combined with non-radioactive thin layer chromatography (TLC) detection was used for screening crude plant extracts for the identification of new 5*α*-R inhibitors.

**Results:**

HHDPCs expressed both 5*α*-R type 1 isoform of the enzyme (5*α*-R1) and AR in all of the passages used in this study. Among the thirty tested extracts, *Avicennia marina* (AM) displayed the highest inhibitory activity at the final concentration of 10 μg/ml, as the production of 5*α*-DHT decreased by 52 % (IC_50_ = 9.21 ± 0.38 μg/ml).

**Conclusions:**

*Avicennia marina* (AM) was identified as a potential candidate for the treatment of AGA based on its 5*α*-R1-inhibitory activity.

## Background

Androgenic alopecia (AGA) is a major type of scalp hair loss in both males and females that is becoming a worldwide issue. AGA is caused by androgens, namely, testosterone (T) and 5*α*-dihydrotestosterone (5*α*-DHT). T is the major circulating androgen and is converted to 5*α*-DHT by the 5*α*-reductase enzyme (5*α*-R) [EC 1.3.99.5], which is present in two isoforms i.e., 5*α*-reductase type 1 (5*α*-R1) and 5α-reductase type 2 (5*α*-R2) [1–6]. Both androgens bind to the androgen receptor (AR), forming a receptor-ligand complex that is translocated to the nucleus, where it acts as a transcription factor in regulating androgen-sensitive genes [7]. However, 5*α*-DHT exhibits five times higher binding affinity and 10-fold higher potency than T in inducing androgen-sensitive genes. The products of these androgen-sensitive genes act as growth factors, affecting hair growth [3, 5, 7–17]. During AGA, overproduction of 5*α*-DHT causes the down-regulation of growth factors, which results in the shortening of anagen/growth phase. This shortening causes the miniaturisation of large, thick-pigmented terminal hair to small, fine, un-pigmented vellus hair with a diameter of less than 0.03 mm [1, 2].

**Fig. 1:**
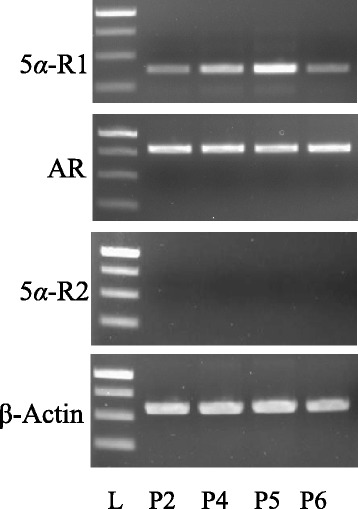
RT-PCR showing the expression of 5*α*-R and AR in HHDPCs. A 1 % agarose gel showing, from the top, the expression of 5*α*-R1 (5*α*-reductase type 1, 380 bp), AR (androgen receptor, 811 bp), 5*α*-R2 (5*α*-reductase type 2, 440 bp) and β-actin (584 bp) within passages 2, 4, 5 and 6 of HHDPCs. The 1-kb DNA ladder (L) shows the band sizes of 1 kb and 750, 500 and 250 bp from top down

Currently, minoxidil and finasteride are the two FDA-approved synthetic drugs used to treat AGA [18]. Minoxidil, a vasodilator and potassium channel opener, prolongs the anagen phase and converts vellus hair to terminal hair [19, 20]. However, minoxidil is only effective on 30-35 % of patients, and the treatment must be continued for life [18]. In addition, the side effects of 2 % and 5 % minoxidil solution include scalp irritation, pruritus, dryness, scaling, itchiness, redness, contact dermatitis and hypertrichosis [21, 22]. Finasteride, a synthetic azo-steroid, is a 5*α*-R2 inhibitor that binds irreversibly to the enzyme and inhibits the conversion of T to 5α-DHT, thereby reducing the serum 5*α*-DHT concentration by 68 % [3]. However, finasteride is effective in only 48 % of the patients [21] and has the observed side effects of impotence, abnormal ejaculation, abnormal sexual function, myalgia, testicular pain and gynecomastia [23].

Because only two synthetic drugs are currently available, a search for new drugs to treat AGA is necessary. Among various sources, natural products provide an abundance of diverse chemical structures, representing a rich source for lead structures in drug development [18]. Recent studies have primarily focused on medicinal plants, and many natural product groups have been demonstrated to have 5*α*-R inhibitory activity. These include a sterol from *Cuscuta reflexa* [24]; fatty acids, including oleic, lauric, myristic and linoleic acid from *Serenoa repens* [25]; a triterpenoid, ganoderic acid from *Ganoderma lucidium* [26]; the saponins soyasaponin I and kaikasaponin III from *Pueraria thomsonii* [27]; and a catechin, epigallocatechin-3-gallate [EGCG] from *Camellia* sinensis [28]. In addition, various well-known flavonoids, such as myricetin, quercitin, alizarin, kaempferol, genistein and daidzein, have also been reported to exhibit inhibitory activity [28]. However, none of these reports have used dermal papilla cells for either their cell-based or enzyme-based (source of 5*α*-R) assay systems. Dermal papilla cells are a primary cell type that regulate hair growth, express 5*α*-R and are the only site of androgen action within the hair follicle [1, 18]. Therefore, the inhibition of 5*α*-R activity in this specific type of cells should have a direct effect on AGA treatment. Therefore, to obtain an inhibitory assay system for this type of hair loss, it is most relevant and reliable to use hair cells, specifically human hair dermal papilla cells (HHDPCs).

In this study, a non-radioactive HHDPC-based assay system, which is highly relevant to the AGA type of human scalp hair loss, was used to screen plant extracts for 5*α*-R inhibitory activity. The most potent crude extract was further tested for the anti-androgenic activity to evaluate its potential to overcome the effects of androgens on the expression of the aforementioned growth factors.

## Methods

### Plant materials and extraction

The list of the Thai plants and plant parts used in this study is shown in Table 1. All plant materials were purchased from of Chao Krom Poe Dispensary in Bangkok, Thailand. Voucher specimens of the plants were deposited in the herbarium collection of Thailand’s Forestry Department with the following specimen numbers (SNs): *Avicennia marina*, SN145917; *Micromelum minutum*, SN055802; *Diospyros mollis*, SN142793; *Alternanthera sessilis*, SN097887; *Salacia verrucosa*, SN099212; *Crotalaria retusa*, SN043656; *Senna timoriensis*, SN094415; *Dalbergia parviflora*, SN200944; *Afgekia sericea*, SN124647; *Bacopa monnieri*, SN176524; *Tarenna hoaensis*, SN176524; *Pterygota alata*, SN059871; *Scoparia dulcis*, SN163093; and *Senna garrettiana*. SN008620. For preparation of crude extracts, the plant part used for each species was ground into powder and extracted through maceration using 100 % methanol at room temperature for two days. The methanolic extracts were then evaporated to dryness at 45 °C using a rotary evaporator (Buchi, Switzerland) and kept at -20 °C until used.

**Table 1:** List of Thai medicinal plants used in this study

Medicinal plant	Abbreviation	Family	Plant part
*Afgekia sericea*	AS	Fabaceae	Aerial
*Alpinia galangal*	AG	Zingiberaceae	Fresh Rhizome
*Alternanthera sessilis*	ASHE	Amaranthaceae	Whole plant
*Avicennia marina*	AM	Acanthaceae	Heartwood
*Bacopa monnieri*	BM3	Plantaginaceae	Aerial
*Balanophora abbreviate*	BA	Balanophoraceae	Aerial
*Barleria cristata*	BC	Acanthaceae	Root
*Butea monosperma*	BM	Fabaceae	Stem
*Centella asiatica*	CA	Mackinlayaceae	Aerial
*Citrus limonum*	CL	Rutaceae	Fresh Peel
*Crotalasia retusa*	CR	Fabaceae	Root
*Dalbergia parviflora*	DP	Leguminosae	Heartwood
*Derris elliptica*	DE	Leguminosae	Stem
*Diospyros mollis*	DM	Ebenaceae	Stem
*Kaempferia galangal*	KG	Zingiberaceae	Dried Rhizome
*Leersia hexandra*	LH	Poaceae	Stem
*Maclura cochinchinen*	MC	Moraceae	Stem
*Micromelum minutum*	MM	Rutaceae	Stem
*Olendra musifolia*	OM	Oleandraceae	Stem
*Pterygota alata*	PA	Malvaceae	Stem
*Randia horrida*	RH	Rubiaceae	Root
*Salacia verrucosa*	SV	Celastraceae	Stem
*Scoparia dulcis*	SD	Plantaginaceae	Stem
*Senna garretiana*	SG	Fabaceae	Heartwood
*Senna timoriensis*	ST	Fabaceae	Stem
*Tarenna fragans*	TF	Rubiaceae	Leaves
*Tarenna hoaensis*	TH	Rubiaceae	Stem
*Telosma minor*	TM	Asclepiadaceae	Stem
*Zanthoxylum limonella*	ZL	Rutaceae	Stem
*Zingiber officinale*	ZO	Zingiberaceae	Fresh Rhizome

### Chemicals, enzymes and reagents

All of the organic solvents used were analytical grade and purchased from RCI Labscan (Bangkok, Thailand). Ultrapure grade dimethyl sulfoxide (DMSO) was purchased from Ameresco® (Framingham, USA). Testosterone (T) and 5*α*-dihydrotestosterone (5*α*-DHT) were purchased from Sigma-Aldrich (St. Louis, USA). Dutasteride was purchased from BDG Synthesis (Wellington, New Zealand). Agarose-LE was purchased from Affymetrix (Santa Clara, USA). Mesenchymal stem cell medium and its supplements were purchased from Sciencell Research Laboratories (Carlsbad, USA). Foetal bovine serum, 100X antibiotic-antimycotic solution, 10X PrestoBlue®, RPMI medium, 50X Tris-acetate-EDTA (TAE) buffer, 0.25 % trypsin-EDTA and Platinum® *Taq* polymerase were purchased from Invitrogen (Grand Island, USA). A GeneRuler 1-kb DNA ladder was purchased from Thermo Fisher Scientific (Pittsburgh, USA). RNeasy® mini kits were purchased from Qiagen (Valencia, USA). DNase I enzyme, first-strand cDNA synthesis kit, dATP, dTTP, dCTP and dGTP were purchased from Fermentas (Walthan, USA).

### Culturing of human hair dermal papilla cells

HHDPCs, obtained from Sciencell Research Laboratories (Carlsbad, USA), were grown in mesenchymal stem cell medium containing 5 % foetal bovine serum (FBS), mesenchymal stem cell medium supplement and 1X antibiotic-antimycotic solution at 37 °C in 5 % CO_2_. The cells between passages 2 to 6 were used in this study.

### Evaluating the presence of 5*α*-R and AR in HHDPCs

Reverse-transcriptase polymerase chain reaction (RT-PCR) was used to identify the type of 5*α*-reductase enzymes (i.e., 5*α*-R1 and/or 5*α*-R2) and AR, expressed in passages 2, 4, 5 and 6 of HHDPCs. Total RNA was extracted from HHDPCs according to the manufacturer’s instructions for the RNeasy® mini kit. The RNA was subsequently treated with DNase I to remove any genomic DNA contamination. Complementary DNA (cDNA) was synthesised from the treated RNA using the first-strand cDNA synthesis kit. The cDNA obtained from this reaction was used as a DNA template for PCR reactions. The PCR reaction comprised of 1X PCR buffer, 2 mM MgCl_2_, 0.4 mM dNTP mix, 0.4 μM each of forward and reverse primer and 2.5 units of Platinum® *Taq* polymerase. The forward and reverse primers for the two isoforms of 5*α*-R (5*α*-R1 and 5*α*-R2) and AR, shown in Table 2, were designed based on the protein region of the full-length sequence obtained from the NCBI GenBank using Clone manager (Scientific & Educational Software, USA) and made to order at 1^st^ Base Laboratories (Selangor, Malaysia). The PCR-thermal profile started with an initial denaturation at 94 °C for 3 min, followed by 35 cycles of denaturation at 94 °C for 30 s, annealing for 30 s at 52 °C, and extension at 72 °C for 2 min, followed by a final extension at 72 °C for 10 min. The PCR products were analysed using 1 % agarose gel electrophoresis.

**Table 2:** Forward and reverse primers and expected sizes of AR, 5*α*-R enzymes and β-actin

Name	Primer pair	Expected size (bp)
Androgen receptor (AR)	F: 5' CGTGCGCGAAGTGATCCAGAA 3'	811
GenBank:NM_000044.3	R: 5' TGCGCTGTCGTCTAGCAGAGAA 3'	
5*α*-Reductase type 1 (5*α*-R1)	F: 5' ACTGCATCCTCCTGGCCATGTTC 3'	380
GenBank:NM_001047.2	R: 5' GGCATAGCCACACCACTCCATGA 3'	
5*α*-Reductase type 2 (5*α*-R2)	F: 5' AAGCACACGGAGAGCCTGAA 3'	450
GenBank:NM_000348.3	R: 5' GCCACCTTGTGGAATCCTGTAGC 3'	
*β*-actin (internal control)	F: 5' ATGATGATATCGCCGCGCTC 3'	584
GenBank:NM_001101.3	R: 5' GCGCTCGGTGAGGATCTTCA 3'	

### Cytotoxicity testing of methanolic plant extracts on HHDPCs

For the cytotoxicity test, HHDPCs were seeded at a cell density of 1x10^5^ cells/ml onto 96-well plates (100 μl of 10,000 cells/well). After 24 h, the cells were separately treated with 100 μl of 5, 10, 20, and 40 μg/ml of each extract to obtain the final concentrations of 2.5, 5, 10 and 20 μg/ml, respectively, and 1 % DMSO (control). Cell viability was measured at 24 h after treatment using 1X PrestoBlue® reagent in RPMI medium. In the presence of viable cells, PrestoBlue® changes from a non-fluorescent blue colour to a fluorescent purple-pink colour, detected using Multimode Detector DTX 880 (Beckman Coulter®, Indianapolis, USA), a bottom-read fluorospectrophotometer with excitation/emission wavelengths of 535 and 615 nm, respectively. The highest final non-toxic concentration of each extract was then used for further studies.

### Screening of plant extracts for 5*α*-R inhibitory activity using HHDPC-based assay combined with non-radioactive TLC detection

HHDPCs were seeded at a cell density of 1x10^5^ cells/ml onto 96-well plates (100 μl of 10,000 cells/well). After 24 h, the cells were separately treated (in a total volume of 200 μl) with the final concentrations of 0.1 mM T and 0.5 % DMSO (internal control), 0.1 mM T and 0.25-20 μg/ml (non-toxic concentrations of the methanolic plant extracts), and of 0.5 % DMSO as negative control. After 48 h, the culture medium of each treatment was collected in Eppendorf tubes, and the attached treated cells were tested for cell viability using the 1X Prestoblue® reagent in RPMI medium. The remained substrate T and the product 5*α*-DHT formed from the activity of cellular 5α-R were extracted from the culture medium using an equal volume of ethyl acetate. The ethyl acetate layer was dried and reconstituted using 20 μl of methanol and spotted onto a TLC Silica gel 60 F_254_ aluminium plate (Merck, Darmstadt, Germany). The TLC plate was developed using toluene:acetone (8:2) as the mobile phase [9]. The developed TLC plate was dipped briefly in a solution of 42.5 % phosphoric acid and heated at 120 °C for 20 min for the visual detection of 5*α*-DHT at 366 nm using a TLC Reprostar Imager (Camag, Switzerland), and the amount 5*α*-DHT formed was quantitated by scanning the product band of 5*α*-DHT at 366 nm using TLC Densitometer Scanner 3 (Camag, Switzerland) to obtain its value of peak area which was then converted to the amount of 5*α*-DHT using a calibration curve. The calibration curve of 5*α*-DHT was constructed by plotting peak areas and various amounts of 5*α*-DHT which showed linearity from 50 to 500 ng with the coefficient of determination (r^2^) value of 0.9874. The extract of AM displaying the highest potential in inhibiting the 5*α*-R enzyme was studied further to determine its effects on androgen-sensitive genes.

### Statistical analysis

All of the experiments were performed in triplicate, and the data are shown as the means ± SD. One-way ANOVA statistical analysis was used, and a *P-value* <0.05 was considered to be statistically significant.

## Results

### Expression of 5*α*-R1 and AR in HHDPCs

The effect of androgens on hair growth is exerted through the interactions with 5*α*-R and AR in HHDPCs. Therefore, the presence of both the enzyme and the receptor is important for this study, and expression in HHDPCs was evaluated. The RT-PCR analysis revealed that the genes of the Type 1 enzyme, 5*α*-R1, and AR were both expressed in passages 2, 4, 5 and 6 of HHDPCs, whereas the Type 2 gene, 5*α*-R2, was not expressed in any of the passages (Fig. 1). β-actin, used as an internal control, was constantly expressed in all passages.

### Cytotoxicity of methanolic plant extracts on HHDPCs

**Fig. 2:**
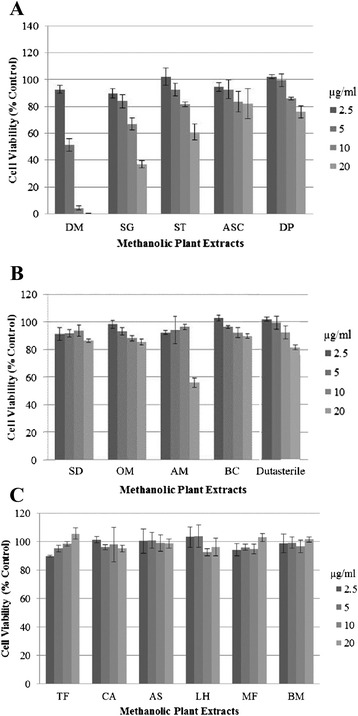
Level of cytotoxicity on HHDPCs of various methanolic plant extracts grouping as (**a**) highest toxicity (2.5 - 5 μg/ml), (**b**) moderate toxicity (>10 μg/ml) and (**c**) low toxicity (> 20 μg/ml)

To obtain a suitable starting concentration for screening methanolic plant extracts for 5*α*-R inhibitory activity, the cytotoxicity of each plant extract on HHDPCs at various concentrations was subsequently tested using Prestoblue® cell viability reagent. The results revealed that the extracts, at their final concentrations, exhibited different toxic levels on the cells (Fig. 2). Above the cell viability of 85 %, the extracts were considered to be non-toxic. The plant extracts of DM and SG were the most toxic, exhibiting toxicity above 2.5 μg/ml, followed by the ST, ASC and DP extracts, which displayed toxicity above 5 μg/ml (Fig. 2a). The other extracts with moderate toxicity above 10 μg/ml were SD, OM, AM and BC (Fig. 2b), whereas the remaining extracts, including TF, CA, AS, LH, MF and BM, displayed no toxicity even at 20 μg/ml (Fig. 2c). Based on these results, the highest final non-toxic concentration of each extract was used for the subsequent inhibitory activity analyses.

### Identification of plant extracts possessing 5*α*-R1 inhibitory activity using an HHDPC-based assay combined with non-radioactive TLC detection

**Fig. 3:**
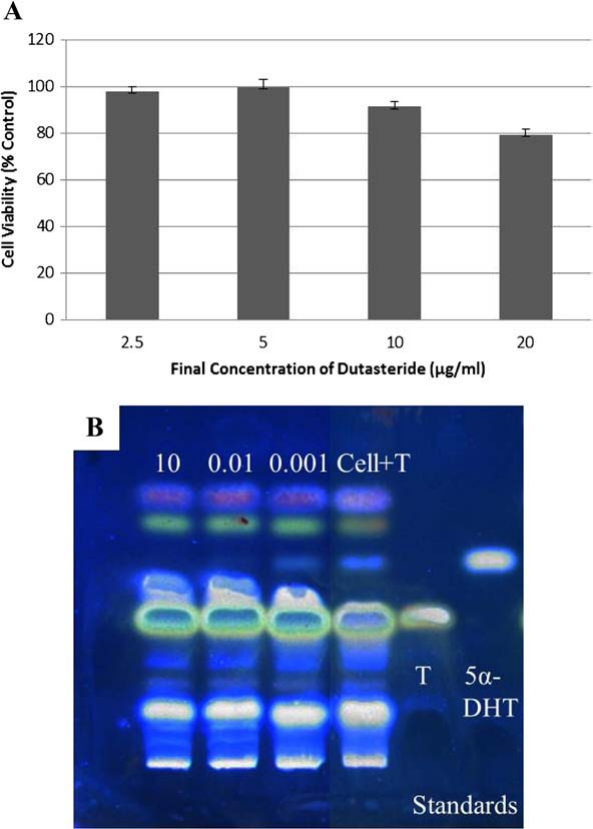
**a** Cytotoxicity at 2.5-20 μg/ml dutasteride and **b** its 5*α*-R1 inhibitory activity at 0.001, 0.01 and 10 μg/ml on HHDPCs showing the inhibition of 16.5 %, 90.5 % and 100 %, respectively

**Fig. 4:**
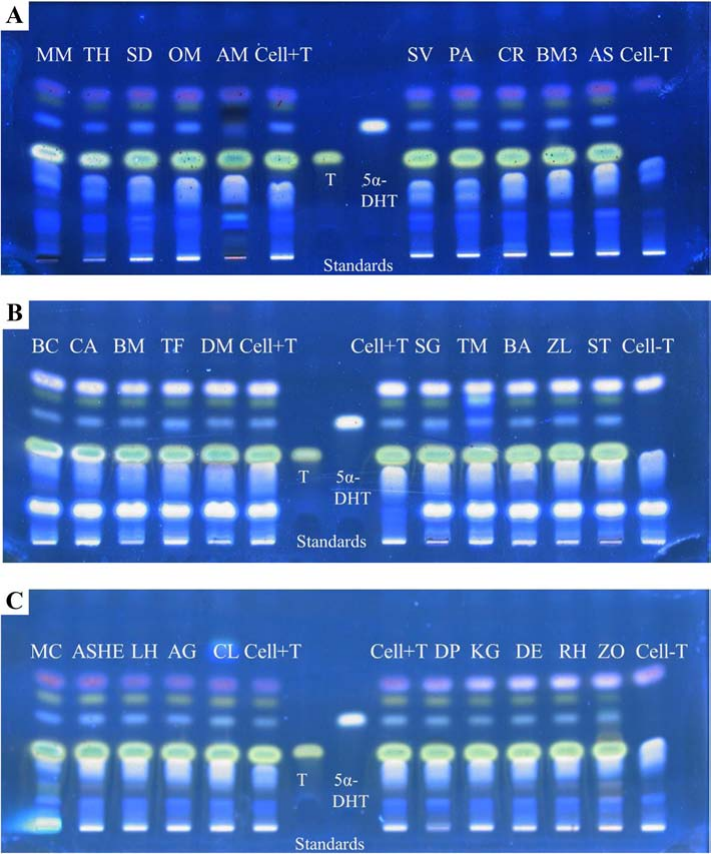
TLC plates visualised under 366 nm showing the effect of 30 methanolic plant extracts on the enzyme activity of 5α-R1. **a** *Micromelum minutum* – MM, *Tarenna hoaensis* – TH*, Scoparia dulcis –* SD, *Olendra musifolia* – OM, *Avicennia marina* – AM, *Salacia verrucosa* – SV, *Pterygota alata* – PA, *Crotalasia retusa* – CR, *Bacopa monnieri* – BM3, *Afgekia sericea*- AS. **b** *Barleria cristata* – BC, *Centella asiatica –* CA, *Butea monosperma* – BM, *Tarenna fragans –* TF, *Diospyros mollis* – DM, *Senna garretiana* – SG, *Telosma minor* – TM, *Balanophora abbreviate* – BA, *Zanthoxylum limonella* –ZL, *Senna timoriensis –* ST. **c** *Maclura cochinchinen –* MC, *Alternanthera sessilis* (hexane extract) *–* ASHE, *Leersia hexandra* – LH, *Alpinia galangal* – AG, *Citrus limonum* – CL, *Dalbergia parviflora* – DP, *Kaempferia galangal* – KG, *Derris elliptica –* DE, *Randia horrida* – RH, *Zingiber officinale* – ZO. The internal (Cell + T) and negative (Cell-T) controls are shown in the middle and right-hand side of the plate, respectively

As previously mentioned, AGA is induced through the over-production of 5*α*-DHT, and the enzyme responsible for the conversion of T to 5*α*-DHT within HHDPCs is 5*α*-R. Thus, one potential method for reducing the effect of the androgens on hair growth is to inhibit this enzymatic reaction. HHDPCs expressed only 5*α*-R1, and therefore, the assay system was tested using a well-known 5*α*-R1 inhibitor, dutasteride, as a positive control. To obtain the starting concentration for testing the inhibitory activity, first, the cytotoxicity of dutasteride for HHDPCs was determined. It was observed that dutasteride exhibited toxicity (i.e., cell viability less than 85 %) above the final concentration of 10 μg/ml (Fig. 3a). At this concentration, the cell-based assay displayed complete 5*α*-R1 inhibitory activity (Fig. 3b). Lowering the concentration of dutasteride to 0.01 and 0.001 μg/ml, the assay displayed the 5*α*-R1 inhibition of 90.5 and 16.5 %, respectively, with an IC_50_ value of 0.005 μg/ml or 9.7 nM (Fig. 3b). The assay system was then used to screen the methanolic plant extracts for 5*α*-R1 inhibitory activity. Using the highest final non-toxic concentration (Table 3) in the incubation mixture, each individual extract was evaluated for this activity. As shown in Figs. 4a-4c, each lane represents the inhibitory potential of each extract at its highest final non-toxic concentration. The lane “Cell + T” was the internal control for the conversion of T to 5α-DHT, and the lane “Cell-T” was the negative control. Among all extracts screened, the plant extracts of BM, DM, OM, MC, BC and BA displayed only 10 % inhibition, whereas the extracts of KG and MM displayed approximately 20 % inhibition. The highest inhibitory activity was observed from the crude extract of AM at a final concentration of 10 μg/ml through the reduction in 5*α*-DHT formation by more than 50 % (Table 3). The IC_50_ value of AM was determined to be 9.21 ± 0.38 μg/ml.

**Table 3:** Percentage of 5α-DHT formation in HHDPCs after being treated with various methanolic plant extracts (at 10 μg/ml) and dutasteride (also at 10 μg/ml). The 5α-DHT formed in the standard bioassay system was set to be 100 %

Plant extracts	Abbreviation	5*α*-DHT formed (% of control)
Internal control	+ Substrate (T)	100 ± 5
(Assay system)	- Substrate (T)	0 ± 0
Positive control	Dutasteride	0 ± 0
*Avicennia marina*	AM	48 ± 3
*Kaempferia galangal*	KG	88 ± 7
*Micromelum minutum*	MM	89 ± 3
*Butea monosperma*	BM	89 ± 5
*Diospyros mollis* ^*a*^	DM	89 ± 4
*Olendra musifolia*	OM	90 ± 9
*Barleria cristata*	BC	91 ± 9
*Maclura cochinchinen*	MC	95 ± 5
*Balanophora abbreviate*	BA	97 ± 4
*Alpinia galangal*	AG	98 ± 3
*Randia horrida*	RH	98 ± 8
*Centella asiatica*	CA	99 ± 1
*Derris elliptica*	DE	99 ± 3
*Telosma minor*	TM	100 ± 4
*Zanthoxylum limonella*	ZL	100 ± 13
*Salacia verrucosa*	SV	101 ± 7.0
*Crotalasia retusa*	CR	101 ± 13
*Senna timoriensis* ^*b*^	ST	102 ± 10
*Citrus limonum*	CL	103 ± 11
*Zingiber officinale*	ZO	104 ± 13
*Dalbergia parviflora* ^*b*^	DP	104 ± 15
*Afgekia sericea*	AS	105 ± 3
*Bacopa monnieri*	BM3	105 ± 9
*Leersia hexandra*	LH	106 ± 2
*Alternanthera sessilis*	ASHE	106 ± 12
*Tarenna hoaensis*	TH	107 ± 12
*Pterygota alata*	PA	110 ± 15
*Scoparia dulcis*	SD	116 ± 4
*Senna garretiana* ^*a*^	SG	116 ± 8
*Tarenna fragans*	TF	117 ± 3

^a^and ^b^the extracts used with the highest non-toxic concentrations at 2.5 and 5.0 μg/ml, respectively

## Discussion

This study focuses on identifying new 5*α*-R inhibitors specifically for treating AGA using a newly developed HHDPC-based assay system. A cell-based assay was chosen over a cell-free assay, where the 5*α*-R enzyme has been reported to be isolated from the rat liver [9, 26, 29], prostate [23, 30], epididymis [27] or the human prostate [24], as the latter does not mimic the actual conditions of the enzymes and does not take into account the toxicity of the test compounds [28]. In addition, the reliability of the cell-free system is presumably low because the sequence similarities between the 5*α*-R1 and 5*α*-R2 from rats and humans are only 61 % and 75 % [6], respectively, which might affect the nature of active site formation and hence the activity. In addition, two isoforms of the enzymes, 5*α*-R1 and 5*α*-R2, are specifically distributed in different organs within the human body [6], and therefore, testing the enzyme isolated from the human prostate [24] might not be relevant to the type of enzyme present in human hair cells.

To obtain a cell-based assay system relevant for hair loss, it is more appropriate to use hair cells than transfected rat [28] or insect [25] cell lines. These cell lines of rats, insects and humans possess different levels of toxicity tolerance to the test compounds, such that a potent concentration of 5*α*-R inhibitor for the rat or insect cell line might be toxic to human hair cells. Hair comprises at least fifteen distinct types of cells where the dermal papilla cell is a primary cell type that regulates hair growth, expresses 5*α*-R and is the only site of androgen action [1, 12, 18]. Therefore, human hair dermal papilla cells (HHDPCs) were used as a model for the identification of potent 5*α*-R inhibitors in this study. After 48 h of T treatment on HHDPCs in our optimised incubation mixture, the enzymatic product, 5*α*-DHT, was detectable and measurable on a TLC plate at 366 nm (Fig. 4) by simply dipping the developed TLC plate into 42.5 % phosphoric acid solution, heating it at 120 °C for 20 mins and quantitatively analysing it using Camag Scan-3 TLC densitometer. The sensitivity of this TLC detection is in the range of ng level (from 25 to 250 ng). Under the normal condition of our cell-based assay which is in the presence of 5.8 μg of T (0.1 mM final concentration), it appears that HHDPCs (10,000 cells) present in the incubation mixture can convert T into 87 ng of 5α-DHT. In the presence of a positive crude extract such as AM, the amount of 5α-DHT formed was reduced to 42 ng, equivalent to 48 % of the conversion (Table 3), which is still in the detectable range.

Therefore, with the high sensitivity of detection, there is no need to use radioactively labelled T and complicated detectors as previously reported, such as a radioactive image analyser [26, 30], TLC radioactive scanners [28], HPLC radioactive detectors [25] or measuring the decrease in radiolabelled T concentration at 254 nm using HPLC [9, 23, 24, 27].

The RT-PCR results revealed the expression of only 5*α*-R1 (Fig. 1), which is consistent with the results of previous studies [6, 31–33]. Therefore, the non-radioactive HHDPC-based assay system was first evaluated with a positive control, dutasteride, which is a well-known specific 5*α*-R1 inhibitor [6]. The compound demonstrated complete inhibition of the enzyme activity at a final concentration of 10 μg/ml with an IC_50_ value of 9.7 nM (Fig. 3). The assay system was then used for screening thirty methanolic plant extracts for 5*α*-R1 inhibitory activity. Among the tested extracts (Fig. 4a-c), it appeared that only the heartwood methanolic extract of *Avicenna marina* (AM) at the final concentration of 10 μg/ml exhibited the highest potential for inhibiting the enzyme activity, as the 5*α*-DHT production was reduced to 48 % (Table 3). The rest of the crude extracts were found to have low or negative inhibitory effect (i.e., ranging from -10 to 20 %) compared with the internal control. To avoid false positive results, the attached treated cells in the 96-well plate were tested for their viability. The cells treated with 10 μg/ml of AM and 10^−4^ M of T for 48 h displayed 100.5 ± 2.02 % (*n* = 3) viability relative to the internal control, confirming the positive effect of the AM extract.

For information about *Avicenna marina* (AM), this plant is commonly called grey or white mangrove. It is a species of mangrove trees belonging to the Acanthaceae family and has been traditionally used in Egypt to cure skin diseases [34]. Phytochemically, terpenoids and steroids, such as lupeol, botulin, β-sitosterol and betutinic acid, have been identified from the bark of AM [34]. Whether any of these compounds are the active 5*α*-R1 inhibitors is still unknown. Therefore, activity-guided fractionation is currently being conducted to identify the active compounds in the AM extract.

## Conclusions

The use of a non-radioactive HHDPC-based assay system in this study has led to the identification of *Avicennia marina* (AM) as a new potential candidate for the treatment of AGA.

## Abbreviations

AGA: Androgenic alopecia
T: Testosterone
5*α*-DHT: 5*α*-Dihydrotestosterone
AR: Androgen receptor
HHDPCs: Human hair dermal papilla cells
5*α*-R: 5*α*-reductase
5*α*-R1: 5*α*-reductase type 1
5*α*-R2: 5*α*-reductase type 2
TLC: Thin layer chromatography
RT-PCR: Reverse-transcriptase polymerase chain reaction. The abbreviations of all plant names used in this study are shown in Table 1
